# Pelvic exenteration with en bloc resection of the pelvic sidewall and intraoperative electron beam radiotherapy with Mobetron^®^ for locally advanced rectal cancer

**DOI:** 10.1007/s10151-017-1649-1

**Published:** 2017-06-14

**Authors:** K. Rangarajan, R. Bhome, N. Bateman, A. Naga, M. Simon, K. Donovan, J. Smith, A. H. Mirnezami

**Affiliations:** 1 Department of Colorectal Surgery, University Hospital Southampton, Tremona Road, Southampton, SO166YD UK; 2 Department of Radiation Oncology, University Hospital Southampton, Southampton, UK; 3 Department of Medical Physics, University Hospital Southampton, Southampton, UK; 4 Department of Radiography, University Hospital Southampton, Southampton, UK; 5 Department of Anaesthesia, University Hospital Southampton, Southampton, UK; 6 Department of Urology, University Hospital Southampton, Southampton, UK; 7 Southampton Complex Cancer and Exenterative Team, University of Southampton Cancer Sciences Division, Cancer Research UK Centre, Somers Cancer Research Building, University Hospital Southampton, Tremona Road, Southampton, SO166YD UK

**Fig. 1:**
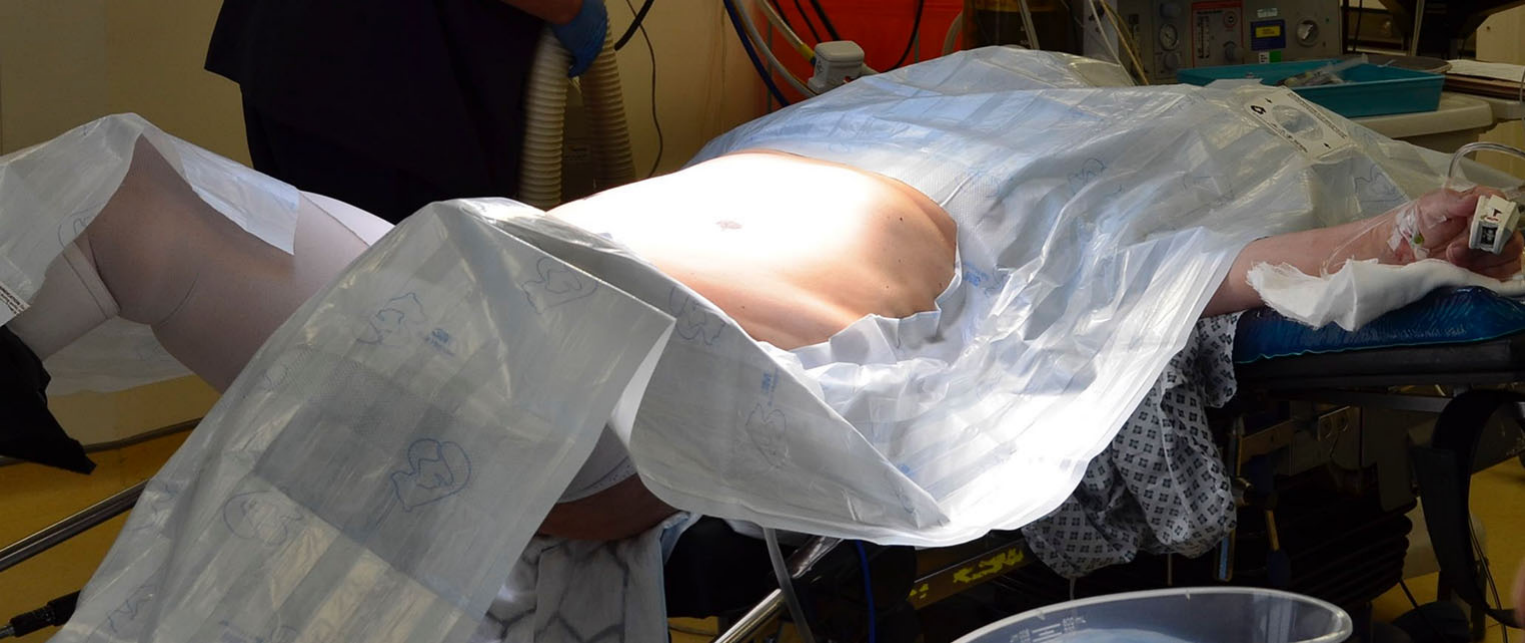
Patient positioning. The modified Lloyds–Davies position is used. Which arm is positioned on the arm board is dependent on the side by which the IntraOp^®^ Mobetron^®^ approaches the patient

**Fig. 2:**
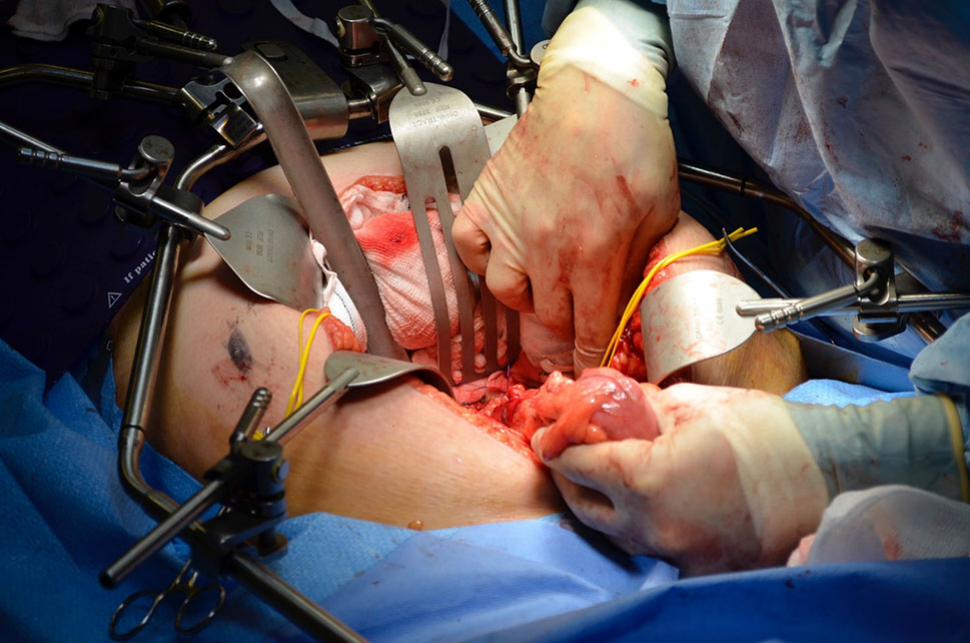
Packing away of small bowel

**Fig. 3:**
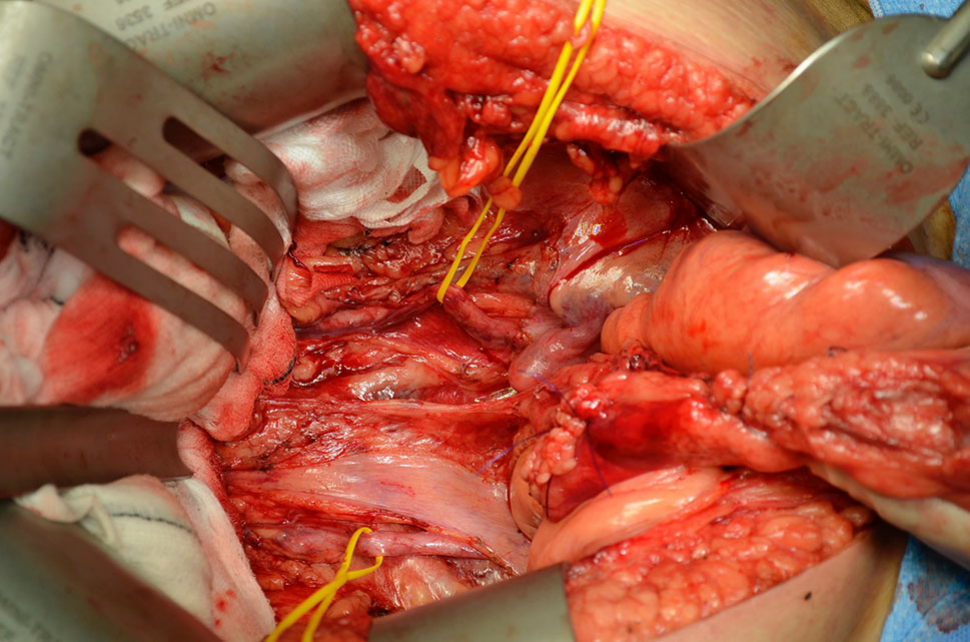
Division of convenience of the colon and identification of the ureters bilaterally

**Fig. 4:**
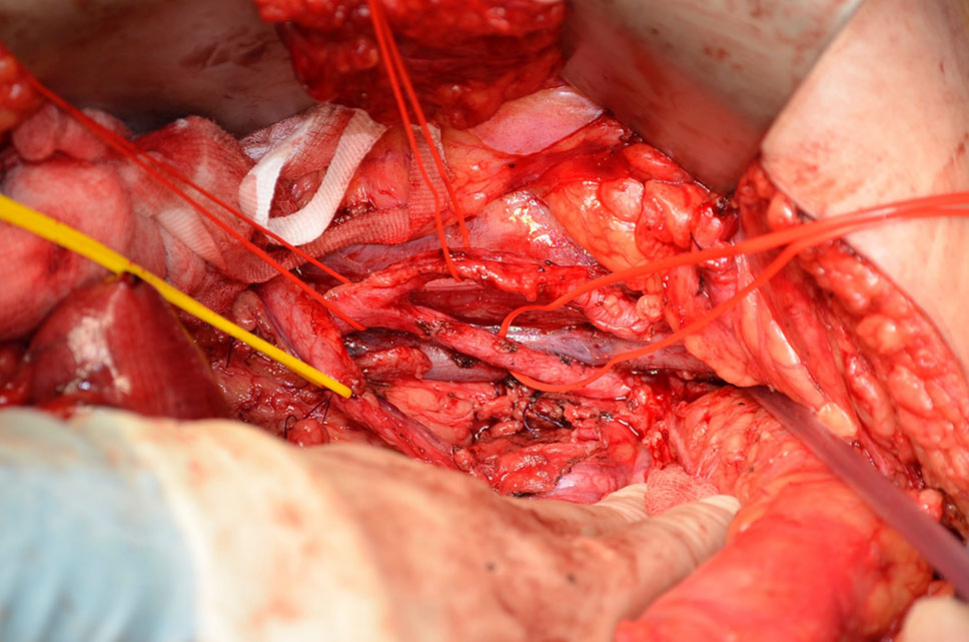
Commencement of pelvic sidewall dissection and identification of the iliac arteries

**Fig. 5:**
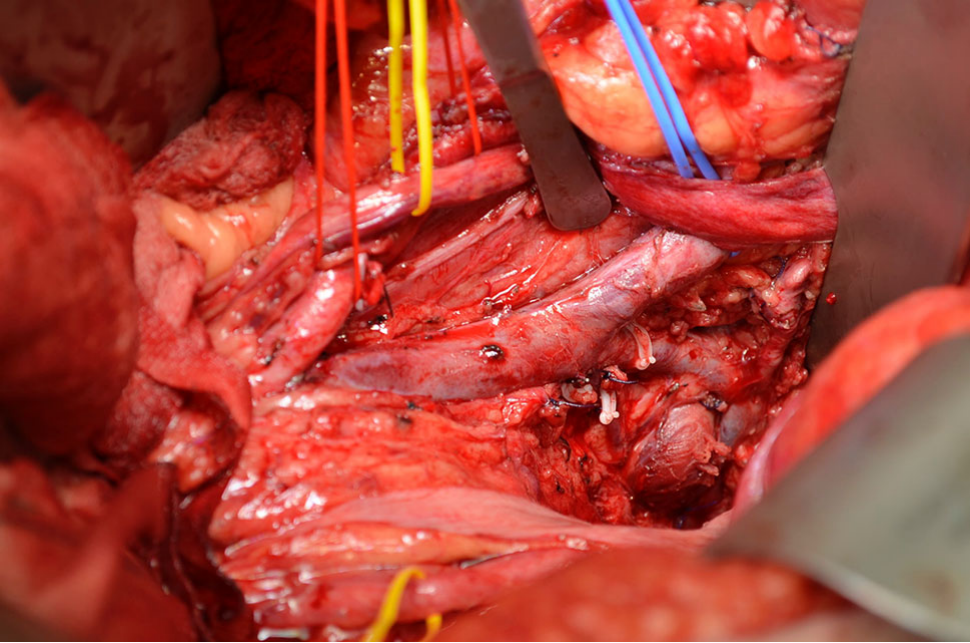
View of pelvis after excision of internal iliac artery and internal iliac vein tributaries with exposure and resection of the fascia over piriformis. This is the radiologically predicted margin of concern

**Fig. 6:**
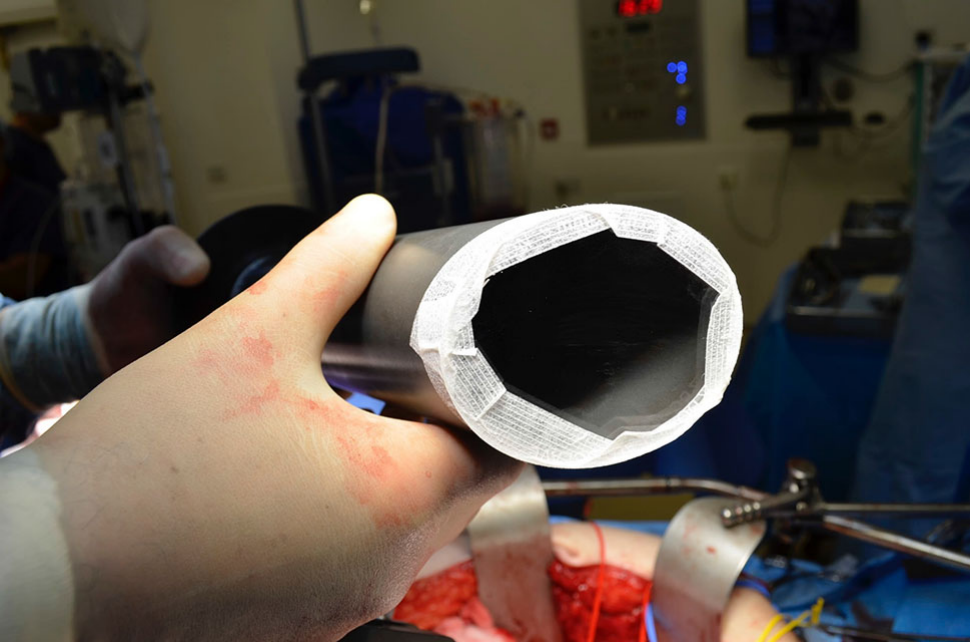
Attachment and securing of the bolus to the intraoperative electron beam radiotherapy applicator (used to modulate dosing in targeted radiation therapy)

**Fig. 7:**
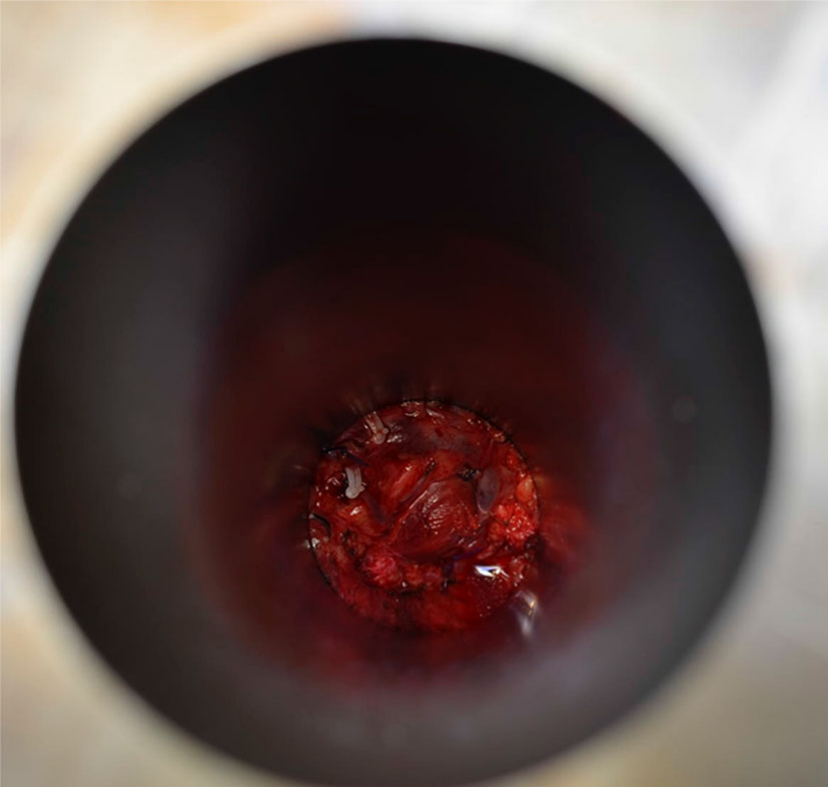
View down the applicator into the pelvis demonstrating the margin of concern within the beam path

**Fig. 8:**
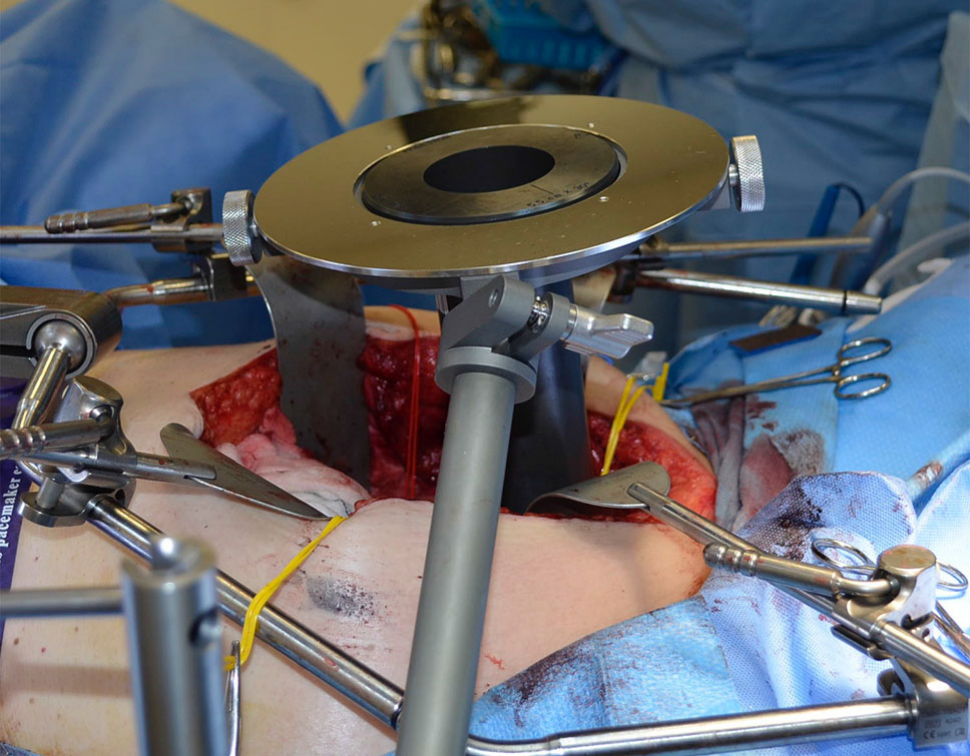
Attachment and positioning of the applicator

**Fig. 9:**
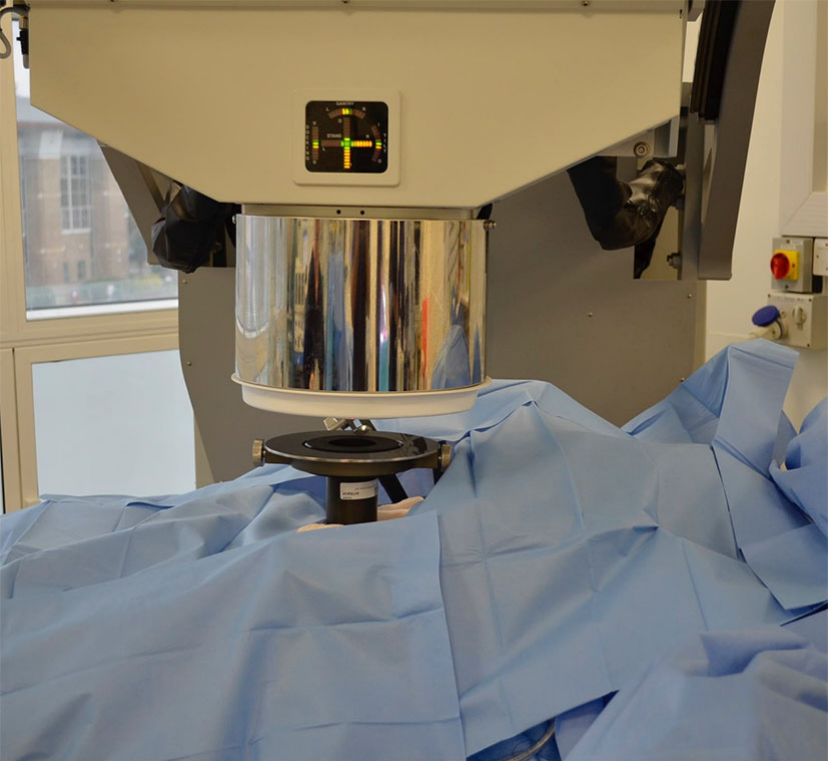
Patient positioning under the IntraOp^®^ Mobetron^®^ mobile linear accelerator device with confirmation of soft docking by laser alignments. 15 Gy intraoperative electron beam radiotherapy was delivered using a 6-cm 30° bevelled applicator

Locally advanced rectal cancer (LARC) is defined as a tumour which is predicted by magnetic resonance imaging to require an extended surgical resection beyond the total mesorectal excision plane [1]. Preoperative neoadjuvant treatments are commonly utilised to downstage and downsize the tumour, facilitating resection. In patients with persistent predicted involved margins or poor response to neoadjuvant treatment, several studies and a meta-analysis have shown that intraoperative electron beam radiotherapy (IOERT) is a further useful adjunct to extended margin surgery, leading to low recurrence within the IOERT field even in patients with a positive margin [2]. Here we describe a case of LARC with anterior and pelvic sidewall involvement and predicted stage of T4N0M0 with involved circumferential resection margins. A poor radiological response to neoadjuvant therapy was noted, and the patient was subsequently treated with a posterior pelvic exenteration with en bloc pelvic sidewall resection and IOERT using the IntraOp^®^Mobetron^®^ device (IntraOp, Sunnyvale, CA, USA). Final histology was ypT4N0 EMVI-negative R1 (pelvic sidewall margin, 0.3 mm) TRG4 (minimal response to neoadjuvant therapy). Post-operative recovery was complicated by a temporary ileus requiring parenteral nutrition. At 6-week and 3-month reviews, the patient had returned to preoperative functional status (Figs. 1, 2, 3, 4, 5, 6, 7, 8, 9).
